# Workplace-based knowledge exchange programmes between academics, policy-makers and providers of healthcare: a qualitative study

**DOI:** 10.1136/leader-2023-000756

**Published:** 2023-07-10

**Authors:** Stephanie Kumpunen, Jake Matthews, Thuvarahan Amuthalingam, Greg Irving, Bernadeta Bridgwood, Luisa M Pettigrew

**Affiliations:** 1 Department of Applied Health Research, Institute of Epidemiology and Health Care, University College London, London, UK; 2 Nuffield Trust, London, UK; 3 Army Medical Services, Camberley, UK; 4 Medcas Education, Birmingham, UK; 5 Faculty of Health, Social Care & Medicine, Edge Hill University, Ormskirk, UK; 6 Department of Cardiovascular Sciences, University of Leicester, Leicester, UK; 7 Department of Health Services Research and Policy, Faculty of Public Health and Policy, London School of Hygiene and Tropical Medicine, London, UK

**Keywords:** multi-professional, career development, health policy, integration, organisational effectiveness

## Abstract

**Background:**

Workplace-based knowledge exchange programmes (WKEPs), such as job shadowing or secondments, offer potential for health and care providers, academics, and policy-makers to foster partnerships, develop local solutions and overcome key differences in practices. Yet opportunities for exchange can be hard to find and are poorly reported in the literature.

**Objectives:**

To understand the views of providers, academics and policy-makers regarding WKEPs, in particular, their motivations to participate in such exchanges and the perceived barriers and facilitators to participation.

**Methods:**

A qualitative study involving semistructured interviews with 20 healthcare providers, academics and policy-makers in England. Rapid data collection and analysis techniques were employed. Interviews formed part of a wider scoping study that mapped the characteristics and existing literature related to WKEPs.

**Results:**

Interviewees reported being motivated to develop, sponsor and/or participate in WKEPs with a clear purpose and defined outcomes that could demonstrate the value of the time out of work to their organisations. Perceived barriers included competitive application processes for national fellowships, a lack of knowing how to identify with whom to undertake an exchange (varying ‘tribes’), and the burdens of time, costs and administration regarding arranging exchanges. WKEPs were reported to work best where there was a perceived sense of shared purpose, long-standing relationship and trust between organisations. Facilitators included existing confidentiality agreements and/or shared professional standards, as well as funding.

**Conclusion:**

WKEPs were reported to be valuable experiences but required significant organisational buy-in and cooperation to arrange and sustain. To benefit emerging partnerships, such as the new integrated care systems in England, more outcomes evaluations of existing WKEPs are needed, and research focused on overcoming barriers to participation, such as time and costs.

**WHAT IS ALREADY KNOWN ON THIS TOPIC**

- Workplace-based knowledge exchange programmes (WKEPs), such as job shadowing or secondments, can foster partnerships and develop local solutions for organisations in the health and care sector. Yet there are few mechanisms for healthcare providers, academics and policy-makers to undertake exchanges.

**WHAT THIS STUDY ADDS**

- People working in health and care are interested in participating in WKEPs and recognise their benefits but have concerns about the organisational effort required to initiate and sustain these programmes.

**HOW THIS STUDY MIGHT AFFECT RESEARCH, PRACTICE OR POLICY**

- More outcomes evaluations and research are needed to support business cases for new programmes, but also to identify routes to overcoming barriers to participation.

## Introduction

 There have been long-standing policy ambitions related to improving the integration of health and care services. The importance of collaborating across professional and organisational boundaries has been reinvigorated by reforms creating integrated care systems (ICSs) across England.^1^ ICSs are area-based structures responsible for planning local services to improve health and care and reduce inequalities in England. They became legal entities in July 2022, with 42 ICSs each covering populations of around 500,000 – 3 million people.^2^ To work cohesively, build relationships and trust within ICSs, member organisations, including hospitals, general practices, community services, local authorities, social care providers, and the voluntary sector organisations, will need to leverage opportunities to learn from each other’s unique experiences and sector knowledge. Yet, at present, there are few mechanisms for people working in these organisations to engage in knowledge exchange activities. Similarly, the desire to improve knowledge exchange between providers, researchers and policy-makers has been well documented, with the aim of improving the effectiveness of the health sector and quality of care provided.

Workplace-based knowledge exchange programmes (WKEPs) provide opportunities for people from different disciplines to spend a temporary amount of time in-person in another workplace, learning from those with differing experience or offering their experience to another organisation.^3^ WKEPs can take varied forms, for example, the types of knowledge exchanged can include scientific/factual knowledge (eg, research findings, quality and performance data), technical knowledge (eg, practical skills, experiences and expertise) and/or practical wisdom (eg, professional judgements, values and beliefs).^4^ WKEPs provide experiential learning opportunities and can include examples such as hospital-based doctors and managers spending time shadowing one another and working together to understand each other’s roles.^5^ WKEPs have a range of aims including: improving knowledge and skills,^6^ fostering deeper partnerships and igniting opportunities for quality improvement,^7^ developing local solutions to practice-based problems,^4^ overcoming key differences in practices, languages, rules between different ‘communities’ (sometimes called the ‘two communities’ problem)^8^ and developing networks or collaborations alongside improving leadership and management skills.^3^ Despite their potential, as illustrated by this list of aims from the literature, very few studies have examined what might help and hinder the development and participation in WKEPs.

Thus, we conducted a qualitative study involving previous participants of WKEPs, to better understand their views on this form of knowledge exchange, as well as their perceptions of barriers and facilitators to both creating and participating in WKEPs. This paper complements another article in which we mapped the characteristics of WKEPs drawing on a scoping review of the academic literature and a systematic mapping exercise of online advertisements of WKEPs in the UK.^3^ In this paper, we were guided by the research questions: *What motivated beneficiaries to start or participate in a workplace-based knowledge exchange programme? What do they believe are the barriers and facilitators of workplace-based knowledge exchange programmes?*

## Methods

This manuscript has been prepared according to COnsolidated criteria for REporting Qualitative research (COREQ) reporting guidelines.^9^ The team involved three academic clinicians, trained in qualitative methods, working in universities at the time of the study, two clinicians working in clinical roles and one trained qualitative health policy researcher. We had each separately taken part in at least one knowledge exchange programme in our careers. We made participants aware of our interest in WKEPs.

A list of organisations in the health and care sector was drafted by the team covering academic, provider and policy-making roles. Fifty-four potential participants from within those organisations were invited via email, which included a study information sheet and consent form. We used purposive and convenience approaches with an aim to interview 20 participants. Where needed, we drew on pre-existing professional relationships to reach people in the selected organisations. Two declined, 21 did not reply to invitations and 31 agreed. We aimed for a balanced sample across organisation types, and the first 20 to agree were included in the sample. The other 11 had been aware of the sampling approach and were notified.

The authors developed a semistructured interview guide (see online supplemental file 1) that aimed to capture the motivations, barriers and facilitators to participation in WKEPs—which complemented the characteristics we mapped in the wider study.^3^ Participants were interviewed via telephone, Zoom or MS Teams between April and May 2020. Verbal consent was recorded at the start of the interview. The length of the interviews ranged between 22 and 70 min.

Interviews were video and audio recorded and transcribed using online software. The data was analysed drawing on principles of rapid analysis,^10^ which included creating, testing and amending a summary template based on the research questions. After each interview, we interpreted interview data and added details in each section of the template including relevant quotes (see online supplemental file 1). We then transferred the template content into an MS Excel matrix of a priori themes, creating tabs for academics, policy-makers and providers, and looked systematically for similarities, differences and trends in responses across groups of informants. To minimise bias, each interview was analysed by at least two researchers. Themes were developed using inductive content analysis in the matrix, discussed among the analysis subteam (SK, JM, TA, LP) and summarised into a longer thematically organised report, which allowed for the iterative identification of key themes and gaps by the whole team.

## Results

The results are presented in four sections, covering interviewees’: (1) demographics and previous experiences of exchanges, (2) appetites and motivations for exchanges, (3) perceived barriers and (4) perceived facilitators of exchanges.

### Participants’ characteristics and WKEP experiences

Most interviewees had multiple roles, six self-identified primarily as academics, seven as policy-makers and seven as providers. Eleven were male and nine were female (see table 1). Fifteen of the 20 participants had management responsibilities, 10 were early or mid-career, and 10 had senior (directorial or executive) roles in their organisations. All interviewees worked in the healthcare sector, rather than social care.

**Table 1:** Participants’ characteristics

	Early career/(first 5 years after postgraduate qualification)	Mid-career	Late career/(last 5 years leading to retirement)
Academics	1 female1 male	1 female	1 female2 males
Policy-makers	2 males	1 female	3 females1 male
Providers	2 males	1 female1 male	1 female2 males

Note: Hospital managers were classed as policy-makers.

Most participants had taken part in organising, facilitating and/or participating in WKEPs involving activities such as:

- Job shadowing during mandated inductions to new roles, semivoluntary shadowing of their teams including managers or receptionists to understand their perspectives, or voluntarily as ‘taster experiences’, for example, to help choose a medical specialty.
- Work placements during undergraduate training and postgraduate qualifications, or voluntary fellowships that involved working/training elsewhere, often as time out of the standard training programme.
- Project-based collaborations, such as quality improvement projects or teaching linked to clinical training, or ad-hoc, self-organised contributions to multidisciplinary team projects done outside of normal working hours.
- Secondments, including spending a temporary period of time in working in another setting or hosting, for example, researchers in policy-based workplaces.

Notably, providers reported limited interaction with national-level policy-makers, whereas all mid-career and late-career academics reported regularly feeding into national-level and regional-level policy-makers.

### Appetite and motivations for exchanges

The majority supported WKEPs, with some arguing the importance of gaining an understanding of peers’ roles and building relationships:

Senior clinicians should be able to read and understand the financial documents required to run their departments. Managers should know the basics of the diseases that affect patients they have in their departments and general pathways. (Provider, P18)

[On secondment] you actually do learn quite a lot. You build relationships and learn about how government works. We don’t teach that that well in universities. So actually, people aren't getting that within the curriculum. (Academic, P5)

While those less supportive of exchanges (the minority) suggested the burdens of coordinating exchange programmes were significant.

I question whether there is any value in bringing all three groups [providers, policymakers and academics] together in a long-term systematic way. Is it really necessary? It’s hard to sustain something over time in a systematic way and people can be brought together on an ad hoc basis. And if done long-term, would there be enough to keep everyone engaged? That would require a standing group with a specific work plan to keep everyone interested. (Academic, P3)

The early-career interviewees discussed their organisations’ senior leaders having reservations about longer WKEPs driven by fear that staff would not return or would be distracted from professional training. However, interviewees who were senior clinicians and managers reported that WKEPs developed leadership skills which were beneficial to the individual and the National Health Service (NHS).

Regardless of career level, most interviewees described a desire for both intradisciplinary and interdisciplinary exchanges at regional and national levels. Some providers described wanting to shadow executives within NHS Trusts, private providers and commissioning organisations. They also reported a desire to take part in training programmes beyond standard requirements and fellowships above and beyond what was required to gain their postgraduate specialisation, such as the Faculty of Medical Leadership and Management’s National Medical Directors Clinical Fellowships scheme. One manager in a hospital suggested a desire to better understand national level policy making, and rhetorically asked, ‘*Who are the people in the Department of Health and what do they do? What’s their background?’ (P20*). Academics and providers described a desire to exchange with national level policy-making organisations including NHS England and the Department of Health and Social Care. One policy-maker suggested value in spending time in *‘any organisation who you work with and need to have a good relationship with but you’re not quite sure how the organisation functions’ (P7*) and their list of examples included: medical colleges, Queens Nursing Institute and cross-discipline bodies (eg, NHS England, General Medical Council; Care Quality Commission). Interviewees based within secondary care and a national research body suggested they wanted better connections with think tanks and the Academic Health Science Networks, as well as with journalists and research funding organisations.

Participants expressed differing goals and motivations for pursuing exchanges. As alluded to above, many participants reported a desire to understand the perspective of others in the sector. Providers reported being motivated by a desire to improve patient care, but also to improve their knowledge, skills and professional networks in order to enhance their career prospects. A mentor–mentee relationship was seen to be particularly advantageous to career progression and to open doors to future projects, funding and positions—exchanges were viewed as a route through which to establish such relationships. Providers reported exchanges helping them standout from their peers when applying for jobs.

Several respondents highlighted the importance of timing and relevance of exchanges impacting their motivations. The perceived ideal timings for participation were often when at a ‘career crossroads’ or when exploring potential career options. Junior clinicians discussed a desire to undertake a unidirectional shadowing experience when deciding whether they wanted to apply for academic or management positions, or which specialty to choose.

A further recurring motivation was for researchers and providers to understand how policy is made. One senior academic leading policy-focused research reported feeling an obligation to provide junior research staff with an understanding of the policy-making process. Providers suggested that policy-makers would benefit from exchanges to the frontline and should seek insight from all the relevant stakeholders affected by policy changes, ‘…*maybe there’s an argument that anybody who’s writing policy on stuff should be accredited as having spent some time on the coalface before they open their mouth and speak about it.’ (Provider, P15*). Exchanges were likewise recognised by policy-makers as important to gaining clinically informed input, from the ‘frontline’, into publications.

### Barriers to exchanges

#### Competition

Early career providers, such as junior doctors, identified barriers of competition being associated with taking part in WKEPs. National fellowship programmes, which often involved prestigious work-placements alongside funding and networking opportunities, were particularly competitive because they required special qualifications or ‘hard-to-obtain experience’. Participants suggested that increased awareness surrounding their importance to career progression had made these types of WKEPs more competitive than in the past.

#### Tribes

Regardless of career stage, half of all participants described a lack of understanding of other professionals’ roles and a lack of awareness of possible ‘partner exchanges’ as a barrier. They reported this lack of knowledge being linked to an inability to communicate between silos because of different terminology, ways of working and timescales of work across disciplines—essentially creating different ‘tribes’. Specific examples provided included clinicians using medical terminology, academics using statistics or research methodology terms, and managers and policy-makers using financial terms or their own acronyms.

#### Administrative

An overarching theme was a perception of bureaucratic hurdles, such as arranging contracts/agreements for WKEP or arranging cover for a clinical shift or completing application forms, grant submissions and travel expenses. Persuading senior managers to agree to release participants for exchanges as well as overcoming subsequent human resources hurdles were seen to require much personal effort. A strong theme was that of negative perceptions from others in their own profession, and especially senior colleagues with regard to ‘wasting time’ not focusing on their own professional specialist development or work. Participants reported that overcoming these administrative hurdles and attitudes involved a great deal of their own time and effort.

#### Cost

At an individual level, WKEPs often required participants to travel or relocate to London, which if coming from elsewhere, often had a negative impact on their standard of living. Funding was provided for structured fellowship programmes which involved work placements or were part of a nationally funded leadership programme, but not for ad hoc job shadowing experiences. In these situations, participants had to make decisions about whether their personal development was worth the investment and they could afford to do so.

From an organisational perspective, to free up a member of staff for an exchange, employing organisations often needed to arrange cover. Early-career providers, in particular, junior doctors, reported that senior colleagues or managers sometimes objected to the exchanges because of the increased financial and administrative load on the department.

#### Time

Providers and managers reported having to plan exchange arrangements and participate in job shadowing and project-focused collaborations in their own time around their day jobs. This was challenging for those on training programmes, where participation and absences are tightly monitored.

Some early and mid-career academics suggested long-term secondments could slow or completely jeopardise career progression by decreasing their output of high-impact publications. However, the senior academic interviewees reported that WKEPs involving work placements or project-based collaborations, including those that were longer term and competitive, tended to accelerate career progression.

### Facilitators of exchanges

Good relationships between leaders and managers were also seen as beneficial for all types of exchanges. Yet, there was also a perception that establishing these relationships was a drain on departmental resources and yielded limited measurable returns. A few participants suggested that their organisation’s participation in a programme was dependent on the exchange having a clear focus and outcomes for individuals and organisations. Provider organisations also suggested that exchanges would need to benefit patients, the individual, sponsoring organisation and the wider NHS. Participants also suggested that when project-focused collaborations needed to be set up quickly, these were enabled by their organisations having a shared purpose (eg, managing the emergent COVID-19 pandemic), as well as long-standing relationships and trust between organisations. Another organisational-level facilitator involved having team members or staff at the fringes of other professional groups or organisations, and thus bridging the divides, facilitating communication, and enabling exchanges.

At an individual level, healthcare staff suggested they were often covered under NHS confidentiality agreements, professional standards and vetted to some degree through their existing employers, enabling them to visit other healthcare or policy-making settings without the need for non-disclosure agreements or significant concern about professional conduct. To facilitate exchanges in clinical settings, interviewees suggested avoiding shadowing inpatient areas or only visiting more controlled environments like outpatient clinics rather than the emergency departments or wards. Finally, having funding to cover exchange expenses was also perceived as an enabler to participation.

## Discussion

We interviewed healthcare providers, academics and policy-makers in England to better understand their experiences of WKEPs and perceptions of the associated barriers and facilitators. Interviewees reported being motivated to develop WKEPs with organisations they worked with but did not understand well, and in particular, those that would help their own organisations achieve their aims. Academics and policy-makers in a sponsorship or leadership position suggested that despite recognising the benefits of exchanges (and having appetite for them), there needed to be a clear purpose for WKEPs and defined outcomes that could demonstrate the value of the time out of work to the organisation. Barriers to participation took the form of competitive application processes, a lack of knowing with whom to undertake an exchange (varying ‘tribes’), administration such as arranging contracts, human resources checks or shift cover in clinical settings, as well as the costs and time involved. WKEPs were perceived to be enabled through organisations having trust and long-standing relationships and a shared sense of purpose or an urgent need for collaboration, such as managing the COVID-19 pandemic, which was emergent during the time of interviews, or examining options for long-term social care funding, which was also a pressing current topic at the time of interviews. Facilitators of WKEPs included having adequate confidentiality agreements and approvals processes, avoiding sensitive or controlled environments during exchanges, and funding for participants to cover expenses.

### Developing the evidence base

Through our wider scoping study, we have sought to coordinate discussions about the characteristics of and literature regarding WKEPs.^3^ However, we have only begun to understand how WKEPs operate and how beneficiaries’ perceptions of their barriers and facilitators might influence the future of WKEPs. Future expansion of WKEPs requires two main factors to be addressed.

There is a need for further evaluation and evidence on WKEPs, providing detailed information on costs and benefits, which can influence employer support of WKEPs. To date, formal evaluations have proved challenging and relatively rare for WKEPs,^3^ this is also seen in the wider topic of ‘knowledge mobilisation’, where other mechanisms to exchange knowledge are used.^11^ One of the main challenges, as identified by our interviewees is that the benefits and knowledge gained appeared to be unique to each exchange and challenging to immediately identify and use. As one of our interviewees suggested *‘no one can teach you in a classroom [what you learn on an exchange]*’ and ‘*you may not actually realise what you’ve learnt until much later’* (*Academic, P11*). Despite these challenges, there is value in developing a framework of outcomes, as well as aiming to comprehensively describe WKEPs in evaluations by drawing on reporting principles which we proposed elsewhere.^3^

Second, there is a need for more research on the benefits and facilitators of WKEPs from the perspectives of experts who have developed them. Our findings on facilitators echo a key message from Oliver *et al*’s study that ‘interpersonal links are important to sustaining knowledge mobilisation, but also need to be underpinned by long-term strategic and institutional support’.^12^ Yet to progress the evidence base, and better support organisational-level business cases, we need to better understand how to overcome barriers, such as how to free up employee time, incentivise participation and identify routes to covering costs.

### Strengths and limitations

The resources available for this project were limited, which meant that we had time for only up to 20 interviews. While we carefully selected interviewees to provide a diverse sample, the research team drew on pre-existing relationships to recruit all but four participants. In addition, we sought out participants with experience in exchanges (and an interest in discussing them), which made for a niche sample of participants who may not represent the norm within the health and social care sector. The sample also did not contain social workers, care workers, nurses, midwives or other allied healthcare professionals—despite our attempts to recruit them. Two focus groups were planned, but the response to the COVID-19 pandemic limited the availability of health and social care staff, and these could not be conducted. Research with a wider sample is advised, including with people within organisations who run WKEPs. Finally, this study examined in-person WKEPs, but there are many different routes to knowledge exchange and knowledge mobilisation, such as embedding researchers in practice through researchers-in-residence,^13^ or communities of practice,^8^ or even leadership development schemes.^14 15^ Some WKEPs pivoted during the pandemic to online and have returned as hybrid models, which suggests there is also value in exploring the differences between modes of operation.

## Conclusion

In this qualitative study on WKEPs, we examined the perspectives of healthcare, policy-makers and academics in England regarding their motivations, perceived barriers, and facilitators of participation in WKEPs. While the appetite for WKEPs was present among all participants, they expressed concerns about the organisational level effort required to initiate and sustain such knowledge exchange programmes. To benefit emerging partnerships and improve integration in the health and care sector, such as through the new ICSs in England, more outcomes evaluations of existing WKEPs are needed, as well as research focused on identifying routes to overcome enduring barriers to participation, such as time and costs.

## Supplementary material

10.1136/leader-2023-000756 online supplemental file 1

## Data Availability

Data are available on request.

## References

[1] Buckingham H, Reed S, Kumpunen S (2023). People, partnerships and place: how can ICSs turn the rhetoric into reality?.

[2] Dunn P, Fraser C, Williamson S (2022). Integrated care systems: what do they look like?.

[3] Kumpunen S, Bridgwood B, Irving G (2023). Workplace-based knowledge exchange programmes between academics, policymakers and providers in the health and social care sector: A Scoping review and mapping exercise. Humanities and Social Sciences Communications - Making and Using Evidence Forthcoming.

[4] Ward V (2017). Why, whose, what and how? A framework for knowledge Mobilisers. Evidence and Policy.

[5] Klaber B, Lee J, Abraham R (2012). Paired Learning: a peer learning Leadership development initiative for managers and clinicians in the NHS.

[6] Bridgwood B, Willoughby H, Attridge M (2017). The value of European exchange programs for early career family doctors. Educ Prim Care.

[7] Aggarwal P, Fraser A, Ross S (2022). Learning from the GP-consultant exchange scheme: a qualitative evaluation. MedEdPublish (2016).

[8] Topp L, Mair D, Smillie L (2018). Knowledge management for policy impact: the case of the European Commission’s joint research centre. Palgrave Commun.

[9] Tong A, Sainsbury P, Craig J (2007). Consolidated criteria for reporting qualitative research (COREQ): a 32-item checklist for interviews and focus groups. Int J Qual Health Care.

[10] Taylor B, Henshall C, Kenyon S (2018). Can rapid approaches to qualitative analysis deliver timely, valid findings to clinical leaders? A mixed methods study comparing rapid and thematic analysis. BMJ Open.

[11] Davies HT, Powell AE, Nutley SM (2015). Mobilising knowledge to improve UK health care: learning from other countries and other sectors - a multimethod mapping study. Health Services and Delivery Research.

[12] Oliver K, Hopkins A, Boaz A (2022). What works to promote research-policy engagement. Evidence & Policy.

[13] Marshall M, Davies H, Ward V (2022). Optimising the impact of health services research on the organisation and delivery of health services: a mixed-methods study. *Health Soc Care Deliv Res*.

[14] Lalleman P, Bouma J, Smid G (2017). Peer-to-peer shadowing as a technique for the development of nurse middle managers clinical leadership: an Explorative study. *Leadersh Health Serv (Bradf Engl*).

[15] Walsh HA, Jolly Inouye AA, Goldman EF (2017). Improving communication through resident-nurse shadowing. Hosp Pediatr.

