# Supplementary material for: Workplace-based knowledge exchange programmes between academics, policy-makers and providers of healthcare: a qualitative study
Source: BMJ Lead. 2023 Jul 10;8(1):e000756. doi: 10.1136/leader-2023-000756 (PMC12038100; doi:10.1136/leader-2023-000756)
Supplement: online supplemental file 1 [file leader-8-1-s001.pdf]

Supplementary File 1

Supplementary Box 1: Interview schedule

Demographic questions:

i. Would you consider yourself an academic, a policymaker, or a health or social care staff member?
ii. Does your role require you to manage a team?
iii. How many years of experience do you have in health and social care research, provision or policymaking?
iv. Have you taken part in an exchange in the past?
v. Would you be open to hosting/facilitating exchanges for your employees in the future?
vi. Interview/Focus Group questions:

Defining the problem and exploring exchanges as a solution

Context: UKRI Research England put out a bid to investigate approaches to improving communication and collaboration between academics and policymakers - which suggests there may be a problem.

1) With that context in mind, have you experienced challenges that have prevented you from collaborating with [policymakers/academics/health and care providers]?
   a) If yes, what specific challenges have prevented you from collaborating with [policymakers/ academics/ health and care providers]? *Probes: Don't know who to contact or how, lack of understanding of their roles, benefits of working with them unclear*
   b) If yes, how have you overcome those challenges?

Your previous exchange experience [If yes to iv]

2) Have you participated, hosted, funded or in any way participated in shadowing, a secondment, a work placement or a sabbatical?
3) In brief, please could you describe your 'exchange' experience? Probes: when? who was involved? what were the aims? where? how long was the exchange? what benefits did you experience?
4) What barriers did you face to participating in this programme?
   a) How did you overcome these barriers?
   b) Have you got any tips on how to reduce these barriers in the future?
5) To what extent could workplace-based exchange programmes help overcome the challenges you listed in collaborating with [policymakers/academics/health and care providers]?
   Mapping exchanges
6) Are you aware of any ongoing exchange/shadowing programmes in the health and social care field between frontline staff, academics and/or policymakers? If so, please give details.
   a) Are you aware of any organisations/ websites/ apps that help to arrange these exchanges? Please describe.

Yes – a future exchange programme is an option [If yes to v]

7) Would you consider participating, hosting or funding a workplace-based exchange?
8) Please could you say more about why you would consider participating, hosting or funding a workplace-based exchange?
   a) What would motivate you / your employer to develop a programme?
   b) Where would the funding for the programme come from?

c) Who might need to be involved or approve of the programme?

9) Which organisations would you ideally like to arrange an exchange with from your current organisation?

10) What barriers would employees face? What barriers would the employer's face? Probes: time, HR, lack of admin help, regulations, shifts, security clearance, travel, not knowing who to contact.

No – an exchange programme is NOT an option [If no to v]

11) Please could you say more about why you would not consider participating, hosting or funding a workplace-based exchange?

Outstanding issues and other potential interviewees

12) Are there any other points you would like to discuss? Is there anyone else you think we should speak to on this topic?

Supplementary Table 1: Interview analysis framework (using a RREAL sheet)

Interviewer:	
Date of interview:	
Participant number(s):	
Demographic questions:	
i. Would you consider yourself an academic, a policymaker, or a health or social care staff member?	
ii. Does your role require you to manage people or a team?	
iii. How many years of experience do you have in health and social care research, provision or policymaking?	
Defining the problem and exploring solutions	
1. Challenges preventing collaboration	
2. Overcoming challenges	
Previous exchange experience	
3. Barriers to previous exchange – and overcoming them (and any relevant info re previous exchange)	
4. Exchanges as a solution	
5. Why participate	
6. Funding and people involved	
7. Ideal exchange partners	
8. Barriers to employee	

9. Barriers to employer	
10. Say why not	
Other	
a. Outstanding questions	
b. Other observations	
c. Mapping exchanges: list and available apps	
d. Important quotations	
